# An Update on COVID-19 Vaccination and Pregnancy

**DOI:** 10.3390/jpm13050797

**Published:** 2023-05-06

**Authors:** Cristina Juliá-Burchés, Alicia Martínez-Varea

**Affiliations:** Department of Obstetrics and Gynaecology, La Fe University and Polytechnic Hospital, Avenida Fernando Abril Martorell 106, 46026 Valencia, Spain

**Keywords:** COVID-19, SARS-CoV-2, COVID-19 vaccine, pregnancy, newborn, immunogenicity

## Abstract

Pregnant women are more prone to experience severe COVID-19 disease, including intensive care unit (ICU) admission, use of invasive ventilation, extracorporeal membrane oxygenation (ECMO), and mortality compared to non-pregnant individuals. Additionally, research suggests that SARS-CoV-2 infection during pregnancy is linked to adverse pregnancy outcomes, such as preterm birth, preeclampsia, and stillbirth, as well as adverse neonatal outcomes, including hospitalization and admission to the neonatal intensive care unit. This review assessed the available literature from November 2021 to 19 March 2023, concerning the safety and effectiveness of COVID-19 vaccination during pregnancy. COVID-19 vaccination administered during pregnancy is not linked to significant adverse events related to the vaccine or negative obstetric, fetal, or neonatal outcomes. Moreover, the vaccine has the same effectiveness in preventing severe COVID-19 disease in pregnant individuals as in the general population. Additionally, COVID-19 vaccination is the safest and most effective method for pregnant women to protect themselves and their newborns from severe COVID-19 disease, hospitalization, and ICU admission. Thus, vaccination should be recommended for pregnant patients. While the immunogenicity of vaccination in pregnancy appears to be similar to that in the general population, more research is needed to determine the optimal timing of vaccination during pregnancy for the benefit of the neonate.

## 1. Introduction

The effectiveness of COVID-19 vaccination was first reported in December 2020. Subsequently, mass vaccination of the general population took place [1]. Nonetheless, pregnant women were not included in initial COVID-19 vaccine trials due to concerns about the lack of prior experience with mRNA vaccines in this population and uncertainty about the potential risks to both the mother and fetus [1,2,3]. As a result, the available information on the long-term safety and efficacy of COVID-19 vaccines in pregnant individuals is currently limited to observational data [1,4]. This limited knowledge has contributed to vaccine hesitancy among pregnant women [1].

Compared to non-pregnant women, pregnant women are at an elevated risk of experiencing severe disease following SARS-CoV-2 infection [1,5,6,7,8]. Indeed, pregnant patients with SARS-CoV-2 infection are three times more likely to require admission to an intensive care unit (ICU), 2.9 times more likely to require invasive ventilation, 2.4 times more likely to need extracorporeal membrane oxygenation (ECMO) and 1.7 times more likely to die [3,5,6,7,8,9,10,11,12,13,14,15,16]. Additionally, pregnant patients are at a higher risk of experiencing sepsis, acute respiratory distress syndrome, thromboembolic events, acute renal failure, and adverse cardiac events compared to non-pregnant patients with COVID-19 [7,12,13,17].

Regarding pregnancy outcomes, pregnant women with SARS-CoV-2 infection are at a heightened risk of preeclampsia/eclampsia, preterm birth, stillbirth, fetal distress, premature rupture of membranes, gestational diabetes, impaired fetal growth, and cesarean section, compared to pregnant women without the infection [7,13,14,15,16,17,18,19,20,21].

Concerning infants, those born to women with laboratory-confirmed SARS-CoV-2 infection are 1.45 times more likely of neonatal adverse outcomes, 1.24 times more likely to require neonatal ICU, 1.61 times more likely to require prolonged neonatal hospitalization and associate a 2-fold higher risk of death, compared to those born without the infection [8,19,22]. The main reason for neonatal morbidity in pregnant women with SARS-CoV-2 infection is prematurity due to the increased rates of preterm deliveries in pregnant women with severe COVID-19 [18].

Given that COVID-19 is associated with maternal, fetal, and neonatal adverse outcomes, the vast majority of scientific societies, including the American College of Obstetricians and Gynecologists (ACOG), the Royal College of Obstetricians and Gynecologists (RCOG), and the World Health Organization (WHO) strongly support COVID-19 vaccination during pregnancy [4]. However, in some countries, COVID-19 vaccination in pregnant women is still not recommended. In Africa, although the vast majority of countries have not been positioned yet, from those who had, Argelia, Libia, and Egypt do not recommend vaccination in pregnant women. In Asia, China, and Kazakhstan, neither recommend the vaccine, whereas, in Europe and South America, it is limited to Lituania and Nicaragüa, respectively [23]. Given that data regarding COVID-19 vaccines in pregnancy is constantly evolving, an update regarding COVID-19 vaccination during pregnancy is presented in this literature review.

## 2. Methods

### 2.1. Search Strategy and Selection Criteria

A literature review was conducted by searching for published studies in the PubMed database written in Spanish or English from November 2021 to 19 March 2023. The search terms used were: (pregnant OR pregnancy) AND (COVID-19 OR coronavirus OR SARS-CoV-2) AND (vaccine OR vaccination) AND (maternal outcomes OR pregnancy complications) AND (neonatal outcomes OR infant complications).

The inclusion criteria were: (1) retrospective or prospective studies that provided a detailed description of the methodological approach used, including cohort studies, case-control studies, case reports, case series, narrative reviews, literature reviews, and meta-analyses, (2) studies with full-text available, (3) studies written in Spanish or English from November 2021 to 19 March 2023, (4) studies that investigated the efficacy and safety of COVID-19 vaccination during pregnancy, (5) studies that addressed the maternal and neonatal impact of COVID-19 vaccines administered in pregnant women, (6) studies that evaluated the immunological patterns of the COVID-19 vaccine in mothers and their neonates during pregnancy. The following studies were excluded: (1) studies that did not provide a detailed description of the methodology used, as well as the number of patients or studies included, (2) editorials or conference papers, (3) studies with no full text available, (4) studies written languages other than Spanish or English, and (5) those irrelevant to the subject of the literature review after reading the title and the abstract.

Additionally, the bibliography of the papers selected was screened in the search for other studies that accomplished the criteria mentioned above to be included. 

### 2.2. Data Extraction

The main objective of this review was to examine the safety of COVID-19 vaccination during pregnancy, including the mRNA-1273 vaccine, the adenovirus vector vaccine, and the BNT162b2 vaccine. The secondary objectives of the study were, firstly, to evaluate the efficacy of COVID-19 vaccines in decreasing unfavorable maternal and neonatal outcomes linked to COVID-19, and secondly, to address whether COVID-19 vaccination during pregnancy enhances the neonatal immune system against SARS-CoV-2 infection either through cord blood or breastfeeding transmission of antibodies. 

The selected studies were analyzed for the following data: (1) basic information about the studies, such as publication year, first author, and study design; (2) characteristics of the study population, including the type of participants, sample size, and location; (3) details about the type of COVID-19 vaccines used, number of doses administered, and timing of vaccination during pregnancy; and (4) results and conclusions of the studies. The basic characteristics of the included studies are summarized in Table 1.

### 2.3. Data Synthesis

A narrative review was conducted using the data gathered from the selected studies. ZOTERO was employed to sort out articles and remove any duplicates. The safety of COVID-19 vaccines during pregnancy was assessed by examining the potential adverse effects described in the literature.

The effectiveness of COVID-19 vaccines in pregnant women vaccinated during or before pregnancy was evaluated based on reported pregnancy outcomes. These included the incidence of SARS-CoV-2 infection, severe illness, hospitalization related to COVID-19, ICU admission, mortality, and other adverse maternal, fetal, or neonatal outcomes. 

Ultimately, data reported in the literature concerning the benefits of maternal COVID-19 vaccination on the neonatal immune system was carefully reviewed.

## 3. Results

### 3.1. Included Articles

**Figure 1:**
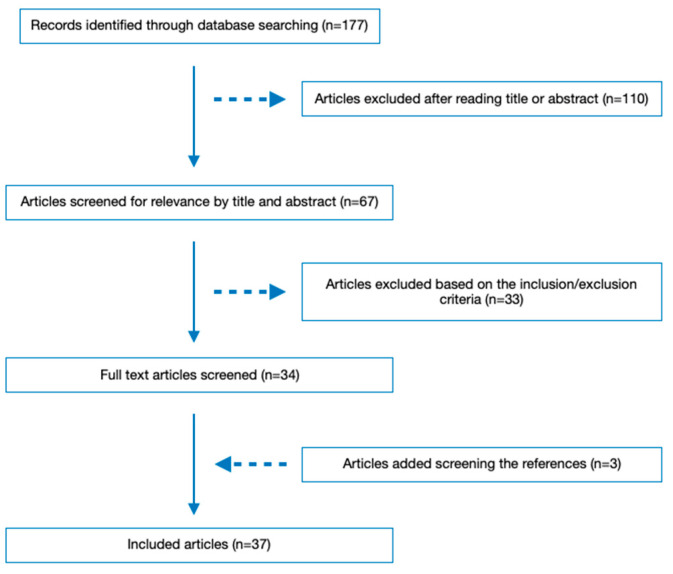
Flow diagram of included articles.

When the initial literature research was conducted up to 19 March 2023, 177 articles were identified. After reviewing the titles and abstracts, 110 articles were excluded. Then, 33 more articles were excluded during the full-text review based on the inclusion and exclusion criteria. Screening the bibliography of the already selected ones, three additional articles were selected. As a result, 37 studies were included in the literature review. The literature retrieval flow diagram is presented in Figure 1. 

### 3.2. Safety of COVID-19 Vaccination during Pregnancy

Observational studies and large case series investigating COVID-19 vaccines in pregnancy have not found any significant adverse events related to the vaccines, apart from the ones commonly described for the general population, such as injection site pain, fever, rash, myalgia, arthralgia, fatigue, headache, chills, lymphadenopathy or lymphadenitis, and nausea or vomiting [24,25]. Moreover, there have been no reports of obstetric, fetal, or neonatal adverse outcomes [1,3,10,24,26,27,28,29,30]. Additionally, there is no evidence of clinically relevant levels of transplacental transfer of mRNA vaccine products [31,32,33].

Hence, there is no proof of an elevated risk of adverse obstetric outcomes, such as miscarriage, preterm birth, earlier gestation at birth, small for gestational age at birth, fetal growth restriction, placental abruption, eclampsia/preeclampsia, gestational hypertension, stillbirth or chorioamnionitis, associated with COVID-19 vaccination during pregnancy [1,11,27,28,34]. Furthermore, COVID-19 vaccination does not appear to increase the risk of birth trauma, mode of delivery, postpartum hemorrhage, maternal death, pulmonary embolism, intensive care unit admission, puerperal fever, the incidence of thromboembolism or length of hospital stay [25,35,36,37,38].

### 3.3. Effectiveness of COVID-19 Vaccines in Reducing Adverse Maternal, Obstetric, and Neonatal Outcomes Associated with COVID-19

Regarding the effectiveness of COVID-19 vaccines in pregnant women, it has been estimated that COVID-19 vaccines are equally effective in pregnant women as in the general population [1,27,39]. In this line, several studies have demonstrated that COVID-19 vaccines prevent the risk of COVID-19 infection, severe COVID-19, hospitalization, ICU admission, and maternal death among pregnant individuals [25,38,39,40,41,42,43,44]. 

Ma Y et al. and Goldshtein et al. reported a 50% reduction in the risk of SARS-CoV-2 infection and COVID-19-related hospitalization with COVID-19 vaccination [10,45]. Other studies have also reported that vaccinated pregnant women have a lower frequency of infection before delivery compared to unvaccinated women [25,39,45]. Moreover, a study published by Kim et al. found that 4.1% and 25% of patients in the vaccination and non-vaccination groups, respectively, had severe symptoms, and 2.6% and 16.2% required oxygen therapy [46]. In this line, Ilter et al. reported in vaccinated pregnant people a significant reduction in the requirement for oxygen support (0.0 vs. 9.6%, *p* = 0.015) and the admission to an intensive care unit (0.0 vs. 3.8%) compared to the unvaccinated group [44]. Furthermore, in a recent study by Eid et al., a significant benefit was found in the vaccinated group with reduced COVID-19 severity, improved clinical results, and fewer hospital or ICU admissions [47].

Regarding obstetric outcomes, Stock et al. and Hui et al. have reported a decrease in stillbirth in pregnant individuals who received the COVID-19 vaccine [39,48]. In addition, a systematic review and meta-analysis found that COVID-19 vaccination was linked to a 15% reduction in stillbirth cases [1]. Watanabe et al. also provided evidence that the administration of COVID-19 vaccines in pregnancy reduced intrauterine fetal death [38].

Concerning preterm delivery, Hui et al. reported a significant decrease in total preterm births <37 weeks (5.1% vs. 9.2%), spontaneous preterm birth (2.4% vs. 4.0%), and iatrogenic preterm birth (2.7% vs. 5.2%) with COVID-19 vaccination [48]. In addition, Carbone et al. and Schrag et al. reported a reduced likelihood of lower gestational age at delivery, non-reassuring fetal monitoring, and a decreased rate of premature delivery in pregnant women who received the COVID-19 vaccine compared to those unvaccinated [43,49].

Table 1 summarizes the main maternal and neonatal benefits and risks of COVID-19 vaccination during pregnancy from the studies included in the review.

**Table 1:** Main maternal and neonatal benefits and risks of COVID-19 vaccination in pregnancy from the studies included in the review.

Main Benefits of COVID-19 Vaccination in Pregnancy	Main Risks of COVID-19 Vaccination in Pregnancy
Reduction in the risk of SARS-CoV-2 infection [10,25,38,39,40,41,42,43,44,45]	Injection site pain [24,25]
Reduction in the risk of severe SARS-CoV-2 infection [25,38,39,40,41,42,43,44,46,47]	Fever [24,25]
Reduction in the risk of COVID-19-related hospitalization [10,25,38,39,40,41,42,43,44,45,47]	Rash [24,25]
Reduction in the risk of ICU admission [25,38,39,40,41,42,43,44,47]	Fatigue [24,25]
Reduction in the risk of maternal mortality [25,38,39,40,41,42,43,44]	Arthralgia [24,25]
Decrease in stillbirth [1,38,39,48]	Myalgia [24,25]
Decrease in total preterm births [43,48,49]	Headache [24,25]
Reduction in the risk of SARS-CoV-2 infection in infants <6 months [50,51]	Nausea or vomiting [24,25]
Reduction in the risk of severe SARS-CoV-2 infection in infants, including MIS-C [3,52]	Chills [24,25]
Reduction in the risk of hospitalization for COVID-19 in infants <6 months [17,25,40,43,53,54]	Lymfadenopathy [24,25]
Reduction in the risk of ICU admission in infants <6 months [3]	Lymfadenithis [24,25]

### 3.4. COVID-19 Vaccination and Potential Benefits for the Neonatal Immune System against SARS-CoV-2 Infection

Regarding hospitalization of newborns, a test-negative and case-control study conducted in 17 US states from July 2021 to January 2022 assessed the efficacy of mRNA vaccines during pregnancy against COVID-19-related hospitalization in infants under six months of age. The study revealed a vaccine effectiveness rate of 61%. Moreover, when administered within the first 20 weeks of gestation, two doses of the vaccine were 32% effective in preventing COVID-19-associated hospitalization in infants, whereas administering the vaccine from 21 weeks gestation through to 14 days before delivery resulted in an efficacy rate of 80% [17,25,40,43,53]. Another study found that maternal vaccination was 52% effective in preventing hospitalization for COVID-19 in infants. The effectiveness was higher at 69% when maternal vaccination was administered above 20 weeks of gestation and lower at 38% during the first 20 weeks of pregnancy [54]. Halasa et al. also pointed out that administering the SARS-CoV-2 vaccine during pregnancy could decrease the risk of infant hospitalization for COVID-19 by 30–70% up to 4–6 months of age [17]. In this line, Kugelam et al. recommend COVID-19 vaccination for pregnant individuals in the second trimester to provide protection for the mother and ensure the safety of the newborn [55]. 

Regarding infection rates, early neonatal SARS-CoV-2 infections were mainly observed among unvaccinated mothers. This indicates that infants born to mothers vaccinated with the mRNA vaccine type had a significantly reduced risk of infection with SARS-CoV-2 compared to infants born to unvaccinated mothers [50,51]. Fully vaccinated mothers had an effectiveness of 61.6% (95% CI, 31.9–78.4), whereas partially vaccinated mothers’ effectiveness was not significant [53]. 

Referring to adverse neonatal outcomes, various studies showed that the administration of the COVID-19 vaccine to pregnant individuals in the third trimester reduced the risk of neonatal adverse outcomes, including multisystem inflammatory syndrome in children (MIS-C) [3,52]. An extensive safety study conducted in Canada indicated that maternal vaccination might protect against low Apgar scores and admission to neonatal intensive care units [43]. Accordingly, Rottenstreich et al. reported that infants born to mothers vaccinated with two doses of mRNA vaccine during gestation had a 30% lower likelihood of COVID-19-associated admission to the ICU in the first six months of life [3]. 

Table 2 summarizes the results from the studies included in the analysis.

## 4. Discussion

The present narrative review has evaluated the impact of SARS-CoV-2 vaccination on maternal, obstetric, and neonatal outcomes. The findings indicate that the uptake of the COVID-19 vaccine during pregnancy does not result in significant adverse events related to the vaccine or poorer outcomes for mothers, obstetric/fetal health, or neonates [1,3,7,10,12,18,24,26,27,28,29,30,57]. In this line, data from the CDC v-safe registry, one of the largest international registries on COVID-19 vaccines during pregnancy, suggest that there are no apparent safety concerns related to COVID-19 vaccination in pregnancy in terms of obstetric or neonatal outcomes [58]. Additionally, the timing of COVID-19 vaccination during pregnancy does not have a significant impact on maternal–fetal outcomes [45]. The available evidence affirms that COVID-19 vaccination during pregnancy is safe and effective, regardless of the gestational age at which it is administered [58]. The main adverse effects of COVID-19 vaccination during pregnancy are the same as in the general population. Therefore, they usually appear within the first seven days, with local complications. Pain at the injection site is the most common one. Regarding systemic complications, fever would be the most frequent and potentially harmful to the fetus. Nonetheless, the use of antipyretics could help to reduce this risk [24,25,59,60]. It is important to note that the COVID-19 vaccine has not been specifically studied in pregnant women who have had complications associated with COVID-19. However, they may benefit from vaccination against COVID-19, as the vaccine can help to reduce the risk of severe illness and adverse outcomes [61]. 

Moreover, the vaccine’s effectiveness appears to be comparable to that observed in non-pregnant individuals [1,5,27,39]. Studies examining vaccine effectiveness have reported varying rates ranging from 41% to 96%, depending on variables including the predominant COVID-19 variant at the time of the study, the vaccine dosage administered, and the outcome variable assessed (severe COVID-19, symptomatic COVID-19, or COVID-19 infection), among other variables [1,27,39]. In general, it is recommended that pregnant women follow the same dosage and administration interval recommendations for COVID-19 vaccination as those applied to the general population. Most COVID-19 vaccines approved so far require two doses, administered at a specific time interval depending on the vaccine type, to achieve maximum efficacy [58,62]. On the other hand, the FDA states that the results from SARS-CoV-2 antibody tests currently authorized should not be used to determine a person’s level of immunity or protection against COVID-19 at any time, including gestation. These tests have not been validated to assess the level of protection, and if results are misinterpreted, there is a risk that individuals may take fewer precautions against SARS-CoV-2 exposure or may or may even choose not to get vaccinated when it is recommended [63].

Regarding maternal benefits of vaccination, the available evidence suggests that pregnant women who are COVID-19 vaccinated experience a lower incidence of infection, severe illness, hospitalization, ICU admission, and need for oxygen therapy prior to delivery when compared to unvaccinated pregnant women [4,5,14,15,25,38,39,40,41,42,43,44,57,64,65]. Additionally, a reduction in preterm birth and stillbirth in vaccinated pregnant women has been proven [1,20,38,48]. It has been reported that stillbirth in pregnant women with COVID-19 is associated with placental insufficiency and infection caused by high levels of viremia [65,66]. Thus, COVID-19 vaccines reduce viremia and may be highly effective in decreasing the risk of stillbirth [67].

Referring to neonates born from vaccinated mothers, studies show a lower rate of SARS-CoV-2 infection, hospitalization, ICU admission, and adverse neonatal outcomes such as MIS-C [3,5,15,17,25,40,43,50,51,52,53,64,65,68]. This benefit to neonates is due to the fetal transfer across the placenta of anti-Spike IgG maternal antibodies induced by the vaccine, which offers stronger protection against SARS-CoV-2 infection during the first few months, with longer disease-free intervals correlated with higher antibodies levels at birth [69]. However, the protection afforded by infant antibodies against SARS-CoV-2 significantly diminishes after six months, highlighting the need for vaccination at this point to maximize defense against COVID-19 [69]. Since COVID-19 vaccines are not authorized for infants under six months, these findings support the notion that newborns benefit from maternal vaccination in terms of protection [25].

Noticeably, maternal vaccination induces a higher antibody response than natural infection, not only through cord blood antibody transmission but also in relation to the transfer of antibodies during breastfeeding [56,70,71]. In fact, breast milk from vaccinated mothers provides immediate protection through antibodies and long-term protection through cellular immunity [72]. Similarly, when referring to transplacental transfer, evidence suggests a direct association between the antibody levels found in maternal serum and cord blood, commonly defined as the placental transfer ratio [25,70,71]. The extent of maternal protection through the transfer of antibodies across the placenta relies on the concentration of antibodies in the maternal bloodstream, which is influenced by the moment of vaccination and delivery, suggesting that the interval between vaccination and birth may be a crucial factor in determining the degree of protection that the mother offers to her newborn [25,56].

Nonetheless, the ideal timing of COVID-19 vaccination in pregnancy to optimize neonatal benefit is still unclear [25,68]. Although the levels of maternal IgG and the transfer ratio of IgG across the placenta tend to rise as pregnancy progresses, the transfer of antibodies through the placenta usually begins during the second trimester, with the highest efficiency observed in the third trimester [25]. Administering the vaccine as soon as possible during gestation is essential to maximize the duration of maternal defense against infection. However, infant protection would depend on the efficiency and concentration of transplacentally acquired antibodies, which most studies suggest is higher during the third trimester [68]. Some studies have reported that all neonatal benefits are higher when mothers are vaccinated after 20 weeks of pregnancy [5,54]. Based on this hypothesis, some experts have recommended administering the booster vaccine in the early third trimester [25]. However, other studies support COVID-19 vaccination before conception or as early as possible during pregnancy to decrease the rate of severe COVID-19 disease during gestation and COVID-19 obstetric complications [40,73]. Nevertheless, at present, given that the majority of pregnant women are immunized against COVID-19, it should be noted that the absolute reduction in the risk after administering a booster dose in this population is likely to be small. Therefore, strategies for the complete vaccination of unvaccinated pregnant women would be more effective in reducing COVID-19 complications than offering booster doses to all those who are already immunized [74,75,76].

This study has several strengths, including its thorough search for evidence, rigorous selection criteria for the relevant literature, meticulous data collection, and objective analysis. Nonetheless, there are also some limitations to mention. Firstly, the retrospective nature of the study design introduces the potential probability of biases that are intrinsic to this type of study. Additionally, unknown factors could influence the findings, such as the impact of different COVID-19 variants and the gestational week at the moment of vaccination, both of which could affect maternal and neonatal outcomes. These factors should be further evaluated in future studies.

## 5. Conclusions

The safest and most effective method for pregnant women to safeguard themselves and their newborns from severe COVID-19 disease, hospitalization, and ICU admission is through COVID-19 vaccination. According to available data, there is no evidence of an increased risk of adverse outcomes for pregnant women, obstetric complications, or neonatal health problems following COVID-19 vaccination. Therefore, vaccination is recommended. The immunogenicity of vaccination in pregnant women appears to be comparable to that of the general population. However, the best timing for vaccination during pregnancy to benefit the newborn is still unclear, underscoring the need for further research.

## Figures and Tables

**Table 2:** Essential characteristics, results, and conclusions of the studies included in the review.

Studies Included	First Author and Year	Type of Study	Participants	Vaccination Data	Results	Conclusions
[1]	Prasad, S. (2022).	Systematic review (23 studies from Israel, USA, UK, Norway, Qatar, and Canada).	n = 117,552 COVID-19 vaccinated pregnant women during pregnancy.	Most studies did not report outcomes according to vaccine type, trimester at vaccination, or number of doses.	The effectiveness of mRNA vaccination against RT-PCR-confirmed SARS-CoV-2 was 89.5% seven days after the second dose (95% Confidence Interval [CI] 69.0–96.4%). The risk of stillbirth in the vaccinated cohort was significantly lower by 15% (pooled Odds Ratio [OR] 0.85, 95% CI 0.73–0.99). There was no indication of an increased risk for other adverse outcomes.	Administering mRNA vaccines for COVID-19 during pregnancy seems to be secure and is linked with a decrease in stillbirth cases.
[3]	Rottenstreich, M. (2022).	Multi-center and retrospective cohort study (Israel).	n = 1750 Pregnant women > 18 years. 712 who received one or two doses of the COVID-19 vaccines during pregnancy, and 1063 unvaccinated.	Vaccinated with Pfizer BioNTech BNT162b2 during the third trimester.	There was no observed association between COVID-19 vaccination and maternal composite adverse outcomes (adjusted OR 0.8, 95% CI 0.61–1.03). A significant decrease in the risk of neonatal composite adverse outcomes was noted (adjusted OR 0.5, 95% CI 0.36–0.74).	Receiving the COVID-19 vaccine during the third trimester of pregnancy did not lead to unfavorable maternal outcomes and, in fact, lowered the likelihood of negative outcomes for the newborn.
[10]	Ma, Y. (2022).	Systematic review and meta-analysis (6 observational studies from USA, Qatar, Israel, and England).	n = 40,978 Pregnant women. 19,108 vaccinated during pregnancy and 21,870 unvaccinated.	Vaccinated with 1 or 2 doses of BNT162b2, Moderna, or adenovirus vector vaccine in any trimester.	Vaccination can help prevent SARS-CoV-2 infection in pregnant women (OR = 0.50, 95% CI, 0.35–0.79) and COVID-19-related hospitalization (OR = 0.50, 95% CI, 0.31–0.82). mRNA vaccines may lower the risk of SARS-CoV-2 infection in pregnant women (OR = 0.13, 95% CI, 0.03–0.57). There were no observed adverse effects of COVID-19 vaccination on pregnant, fetal, or neonatal outcomes.	COVID-19 vaccines appear to be both safe and effective in pregnant individuals.
[11]	Lipkind, H. (2022).	Retrospective cohort study (8 Vaccine Safety Datalink health care organizations from the US).	n = 46,079 Pregnant women aged 16–49 years. 10,064 vaccinated with ≥1 COVID-19 doses during pregnancy, and 36,015 unvaccinated.	Vaccinated with 1 or 2 doses of Pfizer BioNTech, Moderna, or Janssen during any trimester.	There was no observed association between COVID-19 vaccination during pregnancy and preterm birth (adjusted hazard ratio [aHR]= 0.91, 95% CI = 0.82–1.01). There was no observed association between COVID-19 vaccination during pregnancy and small for gestational age (SGA) at birth (aHR 0.95; 95%, CI = 0.87–1.03).	The available data indicate that COVID-19 vaccination during pregnancy is a safe practice.
[17]	Halasa, N. (2022).	Case-control study (US).	n = 379 Hospitalized infants aged <6 months. 176 with COVID-19 (case-infants) and 203 without COVID-19 (control-infants). 148 infants (84%) were born to unvaccinated mothers and 231 infants to vaccinated mothers.	Pregnant women vaccinated with 2 doses of Pfizer BioNTech or Moderna mRNA COVID-19 vaccine at any time during pregnancy.	Maternal vaccination during pregnancy was 61% effective in reducing COVID-19 hospitalization in infants under 6 months of age (95% CI = 31–78%).	Fully vaccinating with two doses of mRNA COVID-19 vaccine during pregnancy may decrease the possibility of COVID-19 hospitalization among infants below 6 months of age.
[22]	Fell, D.B. (2022).	Retrospective cohort study (Canada).	n = 97,590 Pregnant women with mean age 31.9 years. 22,660 (23%) received at least 1 dose of COVID-19 vaccine during pregnancy, and 74,930 (77%) unvaccinated.	Vaccinated with at least 1 dose of BNT162b2, mRNA-1273, or another type of vaccine at any trimester.	There were no significant increases in the risks of postpartum hemorrhage (0.91. 95% CI = 0.82–1.02), chorioamnionitis (0.92, 95% CI = 0.70–1.21), cesarean delivery (0.92, 95% CI = 0.89–0.95), NICU admission (0.85, 95% CI = 0.80–0.90), or low Apgar score (0.84, 95% CI = 0.73–0.97) observed.	There was no significant link found between receiving a COVID-19 vaccine during pregnancy and a higher risk of adverse peripartum outcomes.
[23]	Magnus, M.C. (2022).	Retrospective cohort study (Sweden and Norway).	n = 157,521 pregnant women with a mean age of 31 years (28,506 (18%) were vaccinated against SARS-CoV-2 during pregnancy and 129,015 unvaccinated).	Vaccinated with BNT162b2, mRNA-1273, or AZD1222 at any trimester.	There was no significant association between SARS-CoV-2 vaccination during pregnancy when compared to no SARS-CoV-2 vaccination during pregnancy and the risk of preterm birth, (aHR, 0.98), stillbirth (aHR, 0.86), SGA (aHR, 0.97), low Apgar score (aOR, 0.97), or neonatal care admission (aOR, 0.97).	Receiving the SARS-CoV-2 vaccine while pregnant did not raise the probability of negative pregnancy outcomes.
[24]	Favre, G. (2022).	Observational prospective cohort study (Switzerland).	n = 1012 Vaccinated women during pregnancy.	Vaccinated with at least 1 dose of BNT162b2 Pfizer/BioNTech or mRNA-1273 Moderna in any trimester during pregnancy.	After COVID-19 vaccination, the most commonly reported reactions were local reactions, such as pain at the injection site, and systemic reactions, including fatigue, headache, and muscle pain. Systemic reactions were more frequently reported after the second dose of the vaccine (67.3% vs. 35.3% after the first dose), especially with Moderna mRNA-1273 vaccine. Severe adverse events, such as venous thromboembolism, fever requiring hospitalization, and herpes zoster, were rare following COVID-19 vaccination. It was reported very low rate of early and late spontaneous abortion after COVID-19 vaccination during pregnancy, at 0.9% and 0.4%, respectively. There is no evidence suggesting that COVID-19 vaccination during pregnancy increases the risk of preterm birth or SGA.	Pregnant individuals who were vaccinated did not encounter an elevated risk of negative outcomes during pregnancy or for their newborns.
[25]	Peretz-Machluf, R. (2022).	Retrospective cohort study (Israel).	n = 3700 Pregnant women with a mean age of 32 years. 3240 vaccinated during pregnancy and 460 unvaccinated.	Vaccinated with at least 1 dose of BNT162b2 Pfizer/BioNTech in any trimester.	The administration of COVID-19 mRNA vaccination during pregnancy did not show a significant association with increased risk of adverse obstetric outcomes such as preterm birth, hypertensive diseases of pregnancy, cesarean delivery, and SGA. The vaccinated group had a significantly lower risk of meconium-stained amniotic fluid compared to the unvaccinated group.(aOR 0.63; 95% CI, 0.46–0.86, *p* = 0.0039). There was no significant association found between the COVID-19 vaccine and an increased risk of neonatal adverse outcomes, such as respiratory complications or neonatal intensive care unit (NICU) admission.	There was no significant correlation found between receiving the BNT162b2 messenger RNA vaccine during pregnancy and a higher incidence of negative obstetric or neonatal outcomes.
[26]	Mascolo, A. (2022).	Restrospective case series (Europe).	n = 3252 Individual Case Safety Reports (ICSRs) due to COVID-19 vaccine. 2764 (85%) pregnant women, 258 (7.9%) fetuses/newborns.	Vaccinated with Pfizer-BioNTech, Moderna, Oxford- AstraZeneca, Janssen, or with mixed vaccination at any time during pregnancy.	The incidence of adverse effects following immunization in pregnant women and fetuses/newborns reported in ICSRs was low, with only 0.25% of reports suspecting COVID-19 vaccines as the cause.	No significant indications of previously unknown negative effects linked with COVID-19 vaccination in pregnant individuals have been observed. The advantages of administering COVID-19 vaccines during pregnancy surpass any potential risks.
[27]	Citu, I.M. (2022).	Prospective cohort study (Romania).	n = 835 Pregnant women. 227 vaccinated during pregnancy and 608 unvaccinated.	Vaccinated with BNT162b2 or Ad26.COV2.S during the third trimester.	There were no significant differences in the occurrence of harmful effects between pregnant women and non-pregnant women who received immunization. The examined pregnant women showed a robust humoral response after receiving BNT162b2 and Ad26.COV2.S vaccination.	Both the BNT162b2 and Ad26.COV2.S vaccines are secure to administer during the third trimester of pregnancy, with their effectiveness, safety, and immune response comparable to that of the general population.
[28]	Prahl, M. (2021).	Prospective cohort study (US).	n = 30 Pregnant women vaccinated during pregnancy and their infants.	Vaccinated with BNT-162b2 (Pfizer-BioNTech) or mRNA-1273 (Moderna) at any time during pregnancy.	There is currently no evidence that mRNA vaccine products are present in maternal blood, placental tissue, or cord blood at delivery. The transfer of IgG and neutralizing antibodies from vaccinated pregnant women to their neonates is efficient and time-dependent, with the antibodies persisting during early infancy.	Administering mRNA vaccines during pregnancy is a secure practice and produces time-dependent functional, protective antibody responses in mothers and their infants, which remain effective during early infancy.
[29]	Juttukonda, L.J. (2022).	Prospective cohort study (US).	n = 34 Decidual biopsies obtained at delivery. Eight women had COVID-19 in the first trimester, 17 women were fully vaccinated with 2 doses against COVID-19 during pregnancy, and 9 were neither infected nor vaccinated during pregnancy.	Pregnant women vaccinated with mRNA-1273 (Moderna) or BNT162b2 (Pfizer) at any time during pregnancy.	Lower levels of macrophages and natural killer cells (NK) were observed in the vaccinated cohort compared to the control group. There were no significant differences in cytokine levels between the vaccinated and control groups.	There was no correlation observed between vaccination and inflammation of the decidual tissue, indicating the safety of administering SARS-CoV-2 vaccines during pregnancy.
[30]	Otero, S. (2022).	Prospective cohort study (US).	n = 351 Pregnant women. 252 with SARS-CoV-2 infection, 99 who received COVID-19 vaccination during pregnancy and their 357 infants.	Vaccinated with at least 1 dose of Pfizer NT162b2, Moderna mRNA-1273 or unknown type during the 2nd or 3rd trimester.	Maternal and cord blood IgG levels were found to be higher in pregnant individuals with a more severe SARS-CoV-2 infection category (*p* = 0.0001). The median IgG transfer ratio was 0.87–1.2. Cord blood and maternal IgG were higher after vaccination than infection (*p* = 0.001). In the vaccinated group, the transfer ratio was higher after 90 days (*p* < 0.001). Modeling studies have shown that maternal IgG following vaccination has a higher amplitude and longer half-life compared to maternal IgG produced after SARS-CoV-2 infection (*p* < 0.0001).	Receiving COVID-19 vaccination while pregnant results in greater and longer-lasting levels of maternal IgG antibodies, higher levels of cord blood IgG, and a higher transfer ratio 90 days after vaccination, in contrast to contracting SARS-CoV-2 infection. Increased severity of infection results in elevated levels of both maternal and cord blood antibodies. Maternal IgG levels decline over time after both vaccination and infection, underscoring the necessity of administering COVID-19 vaccines to pregnant individuals, even after infection, as well as administering vaccine boosters.
[31]	Pratama, N.R. (2022).	Systematic review (13 observational studies from US and Israel).	n = 48,039 Pregnant women vaccinated during pregnancy.	Vaccinated with mRNA vaccines (Pfizer–BioNTech and Moderna) at any time during pregnancy.	mRNA-based COVID-19 vaccines can provide strong protection against SARS-CoV-2 infection, without evidence of clear harm in pregnancy. After the first vaccine dose, antibody responses were rapid. The administration of a booster dose after the initial COVID-19 vaccine series resulted in even stronger antibody responses, and this was associated with improved transplacental transfer of antibodies. The longer interval between the first dose of vaccination and delivery was associated with higher levels of fetal IgG and a more favorable antibody transfer ratio.	mRNA vaccines have the potential to decrease the likelihood of future SARS-CoV-2 infections. mRNA vaccines can elicit antibody responses in pregnant individuals and their fetuses Administering two doses of the vaccine to pregnant individuals is recommended to promote stronger antibody responses in both the mother and the fetus.
[32]	Badell, M.L. (2022).	Systematic review (83 studies from the US, Noway, Israel, Romania, Pakistan, England, and Canada).	n = not reported Vaccinated women during pregnancy.	Vaccinated with any type of COVID-19 vaccine at any time during pregnancy.	Based on the available data, there is no evidence to suggest that COVID-19 vaccination during pregnancy is associated with an increased risk of adverse outcomes.	For pregnant individuals, COVID-19 vaccination is the safest and most effective means of safeguarding themselves and their infants against severe COVID-19 illness. The immune response elicited by vaccines in pregnant individuals appears to be comparable to that in the non-pregnant population, although the ideal timing of vaccination during pregnancy for the benefit of neonates/infants is still unclear.
[33]	Hagrass, A.I. (2022).	Systematic review and meta-analysis (13 studies from Qatar, England, the US, Jerusalem, Brazil, and RCT).	n = 56,428 Women vaccinated during pregnancy.	Vaccinated with any type of COVID-19 vaccine at any time during pregnancy.	Statistically significant difference in the following outcomes was not shown: miscarriage (1.56% vs. 0.3%. relative risk [RR] 1.23; 95% CI = 0.54–2.78), length of maternal hospitalization (mean deviation [MD] 0.00, 95% CI = 0.08–0.08), puerperal fever (1.71% vs. 1.1%, RR 1.04, 95% CI = 0.67–1.61), postpartum hemorrhage (4.27% vs. 3.52%, RR 0.84, 95% CI = 0.65 to 1.09. instrumental or vacuum-assisted delivery (4.16% vs. 4.54%. RR 0.94, 95% CI = 0.57–1.56). Incidence of Apgar score ≤ 7 at 5 min (1.47% vs. 1.48%, RR 0.86, 95% CI = 0.54–1.37) and birthweight (MD 7.14, 95% CI = −34.26–19.99).	Administration of the SAR-CoV-2 vaccine does not affect the risk of miscarriage, length of hospital stay, postpartum fever, postpartum hemorrhage, birth weight, or the occurrence of an Apgar score of ≤7 at 5 min.
[34]	Fell, D.B. (2022).	Retrospective cohort study (Canada).	n = 85,162 Live births and stillbirths. 43,099 (50.6%) related to individuals who received one dose or more of a COVID-19 vaccine during pregnancy and 42,063 unvaccinated.	Pregnant women vaccinated with 1,2 or 3 doses of any type of COVID-19 vaccine at any time during pregnancy.	Vaccination during pregnancy was not associated with any increased risk of overall preterm birth (6.5% among vaccinated vs. 6.9% among unvaccinated, aHR 1.02, 95% CI = 0.96–1.08), spontaneous preterm birth (3.7% vs. 4.4%, aHR 0.96, 95% CI = 0.90–1.03) or very preterm birth (0.59% vs. 0.89%, aHR 0.80, 95% CI = 0.67–0.95). There was no evidence of an increased risk of small for gestational age at birth (9.1% vs. 9.2%, aHR 0.98, 95% CI = 0.93–1.03) or stillbirth (0.25% vs. 0.44%, aHR 0.65, 95% CI = 0.51–0.84) associated with COVID-19 vaccination during pregnancy.	Receiving the COVID-19 vaccine during pregnancy is not linked to an increased risk of preterm birth, SGA, or stillbirth.
[35]	Popescu, D.E. (2022).	Prospective cohort study (Romania).	n = 91 Mother/newborn dyads COVID-19 vaccinated during pregnacy.	Pregnant women vaccinated with 2 doses of BNT162b2 Pfizer/BioNTech at any time during pregnancy.	Administration of the COVID-19 vaccine during different periods of pregnancy can stimulate the production of antibodies, which may provide protection for both the mother and the child. The period between vaccination and birth was found to have an impact on the level of serum and breast milk antibodies.	Vaccination of the mother against SARS-CoV-2 has neonatal advantages due to the transfer of antibodies across the placenta during intrauterine development and through breast milk after delivery.
[36]	Watanabe, A. (2022).	Systematic review and meta-analysis (9 observational studies from Israel, the US, England, Canada, and Sweden).	n = 336,695 Pregnant women. 81,349 vaccinated and 255,346 unvaccinated during pregnancy.	Vaccinated with 1or 2 doses of any type of COVID-19 vaccine at any time during pregnancy.	During pregnancy, COVID-19 vaccination was linked to a decreased risk of admission to NICU (OR 0.88, 95% CI = 0.80–0.97) and infection-related fever (IFD) (OR 0.73, 95% CI = 0.57–0.94), whereas it was not associated with preterm birth (OR 0.89, 95% CI = 0.76–1.04), SGA (OR 0.99, 95% CI = 0.94–1.04), and low Apgar score (OR 0.94, 95% CI = 0.87–1.02). Administration of COVID-19 vaccine during pregnancy was linked with a reduced risk of maternal infection with SARS-CoV-2 (OR 0.46, 95% CI = 0.22–0.93) but not associated with increased risk of cesarean delivery (OR 1.05, 95% CI = 0.93–1.20), postpartum hemorrhage (OR 0.95, 95% CI = 0.83–1.07), or chorioamnionitis (OR 0.95, 95% CI = 0.83–1.07).	Vaccination against COVID-19 during pregnancy was not associated with increased risk of peripartum outcomes, but was linked to a decrease in NICU admission, IFD, and maternal COVID-19 infection.
[37]	Stock, S.J. (2022).	Prospective cohort study (Scotland).	n = 117,190 Pregnant women. 18,399 vaccinated and 98,791 unvaccinated.	Vaccinated with 1,2 or 3 doses of any type of COVID-19 vaccine at any time during pregnancy.	The rate of COVID-19 vaccine coverage among pregnant women was significantly lower compared to the general female population of reproductive age. Unvaccinated pregnant women with COVID-19 had a higher incidence of severe complications, such as critical care admission and perinatal mortality, compared to pregnant women who had received the COVID-19 vaccine.	Vaccinating pregnant women against COVID-19 is crucial in preventing adverse outcomes related to the disease.
[38]	Atyeo, C.G. (2022).	Prospective cohort study (US).	n = 175 Maternal-neonatal dyads.	Pregnant women vaccinated with Ad26.COV2.S, mRNA-1273 or BNT162b2 at any time during pregnancy.	Higher titers and functions against SARS-CoV-2 were found after the application of mRNA vaccines in pregnant women. Pregnant women who received COVID-19 vaccination in the first and third trimesters had enhanced maternal antibody-dependent NK-cell activation, cellular and neutrophil phagocytosis, and complement deposition compared to those who received vaccination in the second trimester. Compared to third-trimester vaccination, first and second-trimester vaccination resulted in higher transplacental transfer ratios.	This study shows that there is a higher concentration of functional antibodies in the umbilical cord and a greater transfer efficiency after receiving the first dose of the vaccine during the first trimester of pregnancy. Administration of a booster vaccine during the third trimester, as well as 6 months post-mRNA vaccines and two months post-Ad26.COV2.S vaccine may further enhance maternal and neonatal immunity for pregnant individuals who received their first dose during the first trimester.
[39]	De Rose, D. U. (2022).	Systematic review (45 studies from Israel, England, the US, Qatar, Italy, Belgium, Germany, Norway, and Poland).	n = 80,006 Vaccinated women during pregnancy. 74,908 pregnant women and 5098 lactating women.	Vaccinated with 1,2, or 3 doses of any type of COVID-19 vaccine at any time during pregnancy.	There were no major side effects reported during the COVID-19 vaccine administration, especially during the second and third trimester of pregnancy and during breastfeeding. Maternal vaccination against SARS-CoV-2 has been shown to result in a good maternal immune response, as well as the transfer of maternal antibodies through cord blood and breastfeeding, which can confer passive protection against the virus in newborns.	The benefits of getting vaccinated against COVID-19 during pregnancy for both mothers and infants are greater than the potential risks. Maternal vaccination has been shown to result in the transfer of maternal antibodies that provide passive protection against SARS-CoV-2 in newborns. Breast milk of vaccinated and infected mothers contains anti-SARS-CoV-2 antibodies, which may provide a protective effect on their newborns and infants.
[40]	Morgan, J. A. (2022).	Retrospective cohort study (US).	n = 10,092 Pregnant women. 1332 fully vaccinated and 8760 incompletely vaccinated or unvaccinated.	Vaccinated with 2 doses of any type of COVID-19 vaccine at any time during pregnancy.	Lower odds of severe or critical COVID-19 (0.08% vs. 0.66%, aOR 0.10, 95% CI = 0.01–0.49) and COVID-19 of any severity (1.1% vs. 3.3%, aOR 0.31, 95% CI = 0.17–0.51) were seen in vaccinated pregnant women.	During the fourth surge of SARS-CoV-2, which was dominated by the Delta variant, pregnant patients who received SARS-CoV-2 vaccination had lower odds of experiencing severe or critical COVID-19, as well as any severity of COVID-19, compared to those who were unvaccinated.
[41]	Schrag, S.J. (2022).	Case-control study (US).	n = 5492 Pregnant women vaccinated and unvaccinated with COVID-19-like illness who underwent SARS-CoV-2 molecular testing 4517 ED/UC encounters and 975 hospitalizations.	Vaccinated with 2 or 3 doses of COVID-19 mRNA vaccine at any time during pregnancy.	Maternal mRNA COVID-19 vaccination, including a booster dose, was found to be effective in providing protection against medically attended COVID-19. After maternal mRNA COVID-19 vaccination, including booster dose, the vaccine effectiveness (VE) estimates were higher against COVID-19-associated hospitalization than against emergency department/urgent care (ED/UC) visits. Additionally, the VE estimates were lower against the Omicron variant compared to the Delta variant. The protection offered by maternal mRNA COVID-19 vaccination decreased over time, particularly during the Omicron variant predominance.	Two and three doses of mRNA vaccines provided high maternal protection against medically attended COVID-19 during the Delta variant period. A booster dose provided higher protection against both emergency department and hospitalization endpoints during the Omicron variant predominance.
[42]	Ilter, P.B. (2022).	Retrospective cohort study (Turkey and London).	n = 135 Pregnant women with PCR-confirmed SARS-CoV-2 infection. 83 fully vaccinated and 52 unvaccinated.	Vaccinated with 2 or 3 doses of any type of COVID-19 vaccine at any time during pregnancy.	In vaccinated pregnancies, all cases of SARS-CoV-2 were either asymptomatic or mild, while in contrast, 9.6% of unvaccinated women experienced moderate or severe SARS-CoV-2 symptoms. Compared to the unvaccinated group, the vaccinated group exhibited a significant reduction in the requirement for oxygen support (0.0 vs. 9.6%, *p* = 0.015). In the unvaccinated group, the rate of admission to the intensive care unit was 3.8%, whereas no cases of admission were reported in the vaccinated group.	Fully vaccinated pregnant women who were infected with SARS-CoV-2 during the Omicron wave experienced milder illness and were less likely to require oxygen supplementation and intensive care compared to unvaccinated pregnant women.
[43]	Goldshtein, I. (2022).	Retrospective cohort study (Israel).	n = 15,060 Pregnant women. 7530 vaccinated and 7530 unvaccinated.	Vaccinated with BNT162b2 mRNA vaccine at any time during pregnancy.	Of the infected women, 83.8% (88 out of 105) in the vaccinated group exhibited symptoms, whereas this proportion was 83.2% (149 out of 179) in the unvaccinated group (*p* ≥ 0.99). The risk of infection was 0.33% in the vaccinated group and 1.64% in the unvaccinated group, resulting in an absolute difference of 1.31% (95% CI = 0.89–1.74), with an aHR of 0.22 (95% CI = 0.11–0.43).	There was a notable decrease in the risk of SARS-CoV-2 infection when comparing individuals who received the BNT162b2 mRNA vaccine to those who did not receive any vaccination.
[44]	Kim, H. (2022).	Retrospective cohort study (Korea).	n = 224 Pregnant women. 185 vaccinated and 39 unvaccinated.	Vaccinated with at least 1 dose of any type of COVID-19 vaccine at any time during pregnancy.	There was a significant reduction in the rates of “moderate-to-severe” disease and the need for oxygen therapy in the vaccinated group as compared to the non-vaccinated group (25.4% vs. 4.1%, *p* = 0.005, 16.2% vs. 2.6%, *p* = 0.025; respectively).	Pregnant women who received at least one dose of vaccination experienced a reduced severity of COVID-19.
[45]	Eid, J. (2022).	Retrospective cohort study (US).	n = 472 Pregnant women who tested positive for COVID-19. 25 vaccinated and 347 unvaccinated.	Vaccinated with at least 2 doses of BNT162b2 (Pfizer-BioNTech) or mRNA-1273 (Moderna) or 1 dose of Ad26.COV2.S (Janssen/Johnson & Johnson) vaccine at any time before or during pregnancy.	In the vaccinated group, none of the patients who later contracted COVID-19 progressed to severe or critical disease, whereas it was 7.2% in the unvaccinated group (*p* < 0.01). Individuals who received the vaccine and later developed COVID-19 were less likely to require hospital admission (1.6% vs. 14.7%, aOR 0.14, 95% CI = 0.22–0.47) compared with unvaccinated ones.	For pregnant individuals who receive a vaccination against SARS-CoV-2 and later experience a breakthrough infection, there is a lower probability of experiencing severe or critical COVID-19 and a reduced need for hospitalization or intensive care unit admissions.
[46]	Hui, L. (2022).	Retrospective multi-center cohort study (US).	n = 32,536 Pregnant women. 17,365 (53.4%) vaccinated and 15,171 (47.6%) unvaccinated.	Vaccinated with at least 1 dose of mRNA COVID-19 vaccine before or during pregnancy.	Compared to unvaccinated women, vaccinated women had a significantly lower rate of stillbirth (0.2% vs. 0.8%, 95% CI = 0.09–0.37, *p* < 0.001). There was a significant decrease in vaccinated individuals in the number of preterm births at less than 37 weeks (5.1% vs. 9.2%, 95% CI = 0.51–0.71, *p* < 0.001), spontaneous preterm birth (2.4% vs. 4.0%, 95% CI = 0.56–0.96, *p* = 0.02), and iatrogenic preterm birth (2.7% vs. 5.2%, 95% CI = 0.41–0.65, *p* < 0.001). Newborns delivered by vaccinated mothers exhibited lower rates of admission to NICU. Vaccinated women did not exhibit a significant increase in the rate of congenital anomalies or birth weight below the 3rd percentile. In comparison to the unvaccinated group, vaccinated women had a significantly lower likelihood of giving birth to an infant with a major congenital anomaly (2.4% vs. 3.0%, 95% CI = 0.56–0.94, *p* = 0.02).	Administering the COVID-19 vaccine during pregnancy resulted in a lower incidence of stillbirth and preterm birth and did not negatively affect the growth or development of the fetus.
[47]	Carbone, L. (2022).	Systematic review and meta-analysis (9 studies from Israel, the US, England, and Romania).	n = 40, 728 Pregnant women. 21,297 (52.3%) vaccinated and 19,431 (47.7%) unvaccinated.	Vaccinated with any type of COVID-19 vaccine at any time during pregnancy.	Although the probability of having a SGA fetus remained similar (OR 0.97, 95% CI 0.85–1.09, *p* = 0.570), vaccinated pregnant women showed a lower chance of experiencing non-reassuring fetal monitoring, a decrease in gestational age at delivery, and a lower likelihood of premature delivery compared to unvaccinated pregnant women.	There is a comparable probability of SGA between pregnant women who received the vaccine and those who did not, and vaccinated pregnant women also experienced a slightly lower incidence of premature delivery.
[48]	Danino, D. (2022).	Retrospective multi-center case-control study (Israel).	n = 464 Infants aged 0–6 months hospitalized. 116 SARS-CoV-2-positive infants (cases) were matched with 348 SARS-CoV-2-negative infants (controls).	Pregnant women vaccinated with at least 1 dose of BNT162b2 vaccine at any time during pregnancy.	Fully vaccinated mothers had an effectiveness rate of 61.6% (95% CI = 31.9–78.4), while there was no significant effectiveness observed for partially vaccinated mothers. The effectiveness was observed to be greater in infants aged 0–2 months compared to those aged 3–6 months. The OR for severe infection in infants born to fully vaccinated mothers was 5.8 times lower than that for infants born to unvaccinated mothers.	Infants under 6 months old whose mothers received at least two doses of the BNT162b2 vaccine during the second or third trimester of pregnancy experienced a 61.6% reduction in hospitalization rates for SARS-CoV-2 infection.
[49]	Halasa, N.B. (2022).	Case-control study (US).	n = 1049 Hospitalized infants of 0–6 months. 537 COVID-19 infants and 512 control infants.	Pregnant women vaccinated with 2 doses of mRNA vaccine at any time during pregnancy.	Maternal vaccination was observed to be 52% effective in preventing hospitalization for COVID-19 among infants (95% CI = 33–65). The effectiveness of maternal vaccination in preventing hospitalization for COVID-19 among infants was 69% (95% CI = 50–80) when the vaccination was administered after 20 weeks of pregnancy. In contrast, the effectiveness was 38% (95% CI = 3–60) when the vaccination was given during the first 20 weeks of pregnancy.	Infants under 6 months of age whose mothers received a two-dose mRNA vaccine had a lower risk of hospitalization due to COVID-19, including critical illness.
[51]	Adhikari, E.H. (2022).	Prospective cohort study (US).	n = 2641 Pregnant women with SARS-CoV-2 infection (vaccinated and unvaccinated).	Vaccinated with 2 doses of any type of COVID-19 vaccine at any time before or during pregnancy.	When compared to the pre-Delta epoch, periods where Delta and Omicron variants were predominant were associated with an increase in infections (incidence rate ratios [IRR] 3.07, 95% CI = 2.46–3.82 and 10.09, 95% CI = 7.42–13.69; respectively). Compared to the pre-Delta infections, Delta predominance was associated with an increase in severe or critical illness during pregnancy (OR 2.93, 95% CI = 1.18–7.69), while Omicron predominance was associated with a decrease in severe or critical illness during pregnancy (OR 0.20, 95% CI = 0.05–0.83). Among infants, 90.6% were born to individuals with non-severe illness; and among infected neonates, 90% were born to unvaccinated individuals.	The prevalence of Delta and Omicron variants was linked to a higher incidence of SARS-CoV-2 infections during pregnancy, especially among unvaccinated individuals. The Delta variant was linked to higher illness severity, while the Omicron variant was associated with reduced illness severity. Early neonatal SARS-CoV-2 infections were predominantly observed in newborns whose mothers were not vaccinated and had non-severe COVID-19.
[52]	Carlsen, E. Ø. (2022).	Retrospective cohort study (Norway).	n = 21,643 Newborns. 9739 (45%) born to women vaccinated and 11,904 to unvaccinated women.	Pregnant women vaccinated with at least 2 doses of mRNA COVID-19 vaccine during the 2nd or 3rd trimester.	Infants born to mothers who were vaccinated during pregnancy had a lower risk of testing positive for COVID-19 compared to infants born to unvaccinated mothers, particularly during the Delta variant-dominant period (IRR 1.2 vs. 3.0 per 10,000 follow-up days; 95% CI = 0.19–0.46) compared to the Omicron period (IRR 7.0 vs. 10.9 per 10,000 follow-up days, 95% CI = 0.57–0.79).	Newborns born to women who received a second or third COVID-19 vaccine dose during their last two trimesters of pregnancy experienced a lower incidence of SARS-CoV-2 infection within the first four months of their life compared to newborns born to unvaccinated women. There was a larger decrease in the risk of infant infection during the period when the Delta variant was predominant, as compared to the period when the Omicron variant was dominant. There is preliminary evidence to suggest that infants may receive passive protection against SARS-CoV-2 infections when their mothers are vaccinated against COVID-19 during pregnancy.
[53]	Mangat, C. (2022).	Narrative review (19 studies from Israel, the US, Italy, Germany, Poland, and Belgium).	n = 50,618 Infants (diagnosed with COVID-19 and not).	Pregnant women vaccinated with any type of COVID-19 vaccine at any time during pregnancy.	The evaluation of the risk of Multisystem Inflammatory Syndrome in Children (MIS-C) in infants born to vaccinated mothers has not been extensive. Vaccination of pregnant individuals results in the production of anti-spike protein IgG antibodies in maternal circulation, which are then passively transferred to the fetus through transplacental transport. These antibodies can be detected in newborns after birth and during early infancy and have a neutralizing effect against COVID-19 infection and its complications, including MIS-C.	Administering the COVID-19 vaccine to pregnant women led to a reduced risk of neonatal adverse outcomes, including MIS-C.
[56]	Martínez-Varea, A. (2022)	Retrospective cohort study (Spain)	n = 487 Pregnant women with SARS-CoV-2 infection. 201 (41.27%) vaccinated and 286 (58.73%) unvaccinated.	Vaccinated with at least 1 dose of any type of COVID-19 vaccine at any time before or during pregnancy.	The probability of testing positive for cord-blood SARS-CoV-2 IgG antibodies was found to be 89% lower when the mother was infected with SARS-CoV-2 during the third trimester of pregnancy, in comparison to being infected during the first or second trimester. (OR 0.112; 95% CI 0.039–0.316). COVID-19-vaccinated pregnant women had an 80% reduced risk of developing pneumonia and being hospitalized for COVID-19 compared to those who were not vaccinated (aOR 0.209; 95% CI 0.044–0.985). Pregnant individuals with SARS-CoV-2 infection that have received two or more doses of the COVID-19 vaccine did not experience severe COVID-19.	Vaccinated women that contracted SARS-CoV-2 infection during pregnancy had lower rates of hospitalization due to COVID-19 and were less likely to develop severe disease than unvaccinated patients.

## Data Availability

Not applicable.
